# Strength of female mate preferences in temperature manipulation study supports the signal reliability hypothesis

**DOI:** 10.1371/journal.pone.0303691

**Published:** 2024-06-06

**Authors:** Nicole E. Cobb, Samantha M. Mason, Keith Tompkins, Meredith Fitschen-Brown, Oscar Rios-Cardenas, Molly R. Morris

**Affiliations:** 1 Department of Biological Sciences, Ohio University, Athens, OH, United States of America; 2 Red de Biología Evolutiva, Instituto de Ecología, Asociación Civil, Xalapa, Veracruz, México; University of Iceland, ICELAND

## Abstract

Both sexually selected traits and mate preferences for these traits can be context dependent, yet how variation in preferred traits could *select* for context dependent preferences has rarely been examined. The signal reliability hypothesis predicts that mate preferences vary across contexts (e.g., environments) in relation to the reliability of the information preferred traits provide in those contexts. Extensive variation in copy number of *mc4r* B alleles on the Y-chromosome that associates with male size in *Xiphophorus multilineatus* allowed us to use a split-sibling design to determine if male size is more likely to provide information about male genotype (i.e., dam) when males were reared in a warm as compared to a cold environment. We then examined strength of preference for male size by females reared in the same two environments. We found that males were larger in the cold environment, but male size was more variable across dams in the warm environment, and therefore male size would be a more reliable indicator of dam (i.e., genetics) in the warm environment. Females reared in the warm environment had stronger mate preferences based on male size than cold reared females, with a significant influence of dam on strength of preference. Therefore, strength of female preference for male size was influenced by the temperature in which they were reared, with the direction of the difference across treatments supporting the signal reliability hypothesis. Understanding how the reliability of male traits can select for contextual variation in the strength of the female mate preferences will further our discovery of adaptive mate preferences. For example, a relationship between the strength of a female’s mate preference and their growth rates was detected in the context where females had a preference based on male size, supporting a hypothesis from previous work with this species of disassortative mating in relation to growth rates to mitigate a documented growth-mortality tradeoff.

## Introduction

The mating decisions of females can have important consequences for their fitness [1]. Investing in being choosy can benefit females through reduced costs of finding a male (e.g., sensory bias), mating with males that provide more resources (i.e., direct benefits) and/or better genes for increasing offspring viability (i.e., indirect benefits). Both sexually selected male traits and female mate preferences are often conditional in their expression, and the study of this variation can provide valuable insights into adaptive mate preferences. If the potential genetic benefits of being choosey are also context dependent, the expression of preferences could temporally be reduced in some contexts (i.e., females would be less choosy) [1]. Another possibility, however, is that the genetic benefits do not change across contexts, but the ability to detect differences across males based on the genetic benefits changes. In this case, there could be selection for females that have stronger mate preferences in the contexts where the traits reveal the most reliable information about the potential benefits of choosing between mates [2].

The relationship between the expression of sexually selected traits and components of fitness has extensive support (for reviews see [3,4]), and the level of heritable variation for trait expression often depends on the degree of condition dependence of such traits (e.g., [5,6]). In some contexts, conditional traits will express more heritable variation [1,7,8]. For example, greater additive genetic variance relative to the sexually selected trait eye span in stalk eyed flies was detected when flies were fed cotton wool as compared to spinach [9]. However, studies that examine how female mate preferences vary across contexts depending on the information that is accessible about a male’s genotype (i.e., reliability) are rare. The signal reliability hypothesis is based on the concept that the environments that produce more variation in a trait between different genotypes are the environments where the trait provides more reliable information about genotype (i.e., indirect benefits). In relation to variation in female mate preference for these traits, the signal reliability hypothesis predicts the evolution of stronger mate preferences in those environments where the preferred traits are more reliable indicators of an individual’s genotype.

*Xiphophorus multilineatus* females prefer larger males on average when given a choice between the larger courter alternative reproductive tactic (ART) males and the smaller sneaker ART males, with the measure of preference in the laboratory reflecting mate choices females made in the field [10]. The ARTs in this species differ in several other traits in addition to body size, including age at sexual maturity [11], body depth, sword length [12], growth rates [13,14], and plasticity in mating behaviors. The sneaker males use both coercive (sneak-chase) and coaxing (courtship) mating behaviors, while the courter males are fixed in their use of courtship behavior regardless of their size [15]. In addition, several different factors have been found to influence female mate preferences in swordtail fishes [16–19], including female’s nutritional environment [20], social context [16], genotype and growth rate [21]. However, previous studies have not considered the possibility of a relationship between the context influencing mate preference and the reliability of the preferred male traits in that context.

Here we determined how rearing temperature influenced both variation in male size in relation to genotype (as assessed by dam) and variation in the strength of female mate preference based on male size in the swordtail fish *X*. *multilineatus*. To avoid confounding differences between the ARTs in this species and focus on male size, we used only males and females from the courter ART lineage. The ARTs breed true in this species due to genetic variation of the *mc4r* gene on the Y chromosome and the extensive variation in male size within the courter male ART is associated with copy number variation of the *mc4r* B alleles [22,23]. Using a full sibling-split experimental design, we addressed two questions. First, does male size provide more information about potential differences in the *mc4r* genotype across courter males in a warm environment as compared to a cold one? Second, is the strength of female mate preference for body size greater when females are reared in the temperature where male size would provide the most reliable information about genotype?

## Methods

### Subjects

All work was approved by Ohio University Institutional Animal Care and Use Committee (IACUC) and we were informed in a written approval (12-L-042). *Xiphophorus multilineatus* has two male alternative reproductive tactics that breed true (ARTs, larger courter males and smaller sneaker males, [12,24]). Twenty-nine females from a breeding mesocosm, set up with 10 virgin females and 10 courter males for over 3 generations in a 284-liter tank, were isolated and allowed to drop fry over spring 2021. After 4 weeks of age, siblings were split between two temperature treatments (20°C and 25°C). Temperatures span the range of what this species experiences in the wild depending on locality [25]. Frequency of multiple paternity in this species is low [26], suggesting fry from the same brood were full siblings. Variation in the number of copies of the *mc4r* B alleles on the Y chromosome is associated with the extensive variation in size within the courter male ART, with relatively small courters having a single copy of the allele, as compared with the largest of the courter males having up to 6 copies of the B allele [22]. We only used fry from females isolated from a courter mesocosm, and therefore all the males were courter males and all the females would have had courter male sires.

Each fish was housed separately in a 21-liter tank with dividers between tanks to remove visual contact with other fish. The number of tanks in each environmental chamber was the limiting factor in relation to sample size. Rooms were kept on a 12:12 hour light:dark cycle, and all fish were fed daily Spirulina-based flake food in the mornings, and live brine shrimp *nauplii* in the afternoons on weekdays. Therefore, we controlled for the two environmental factors other than temperature that are known to influence adult male size (i.e., diet and exposure to other adult males, [27]).

### Morphology and growth rates

Photographs of males at 100 days were used to calculate their early growth rates (mm/day) and at 330 days, after all had reached sexual maturity, to determine adult male size. Growth rates up to 100 days have been found to influence courter male’s probability of reaching sexual maturity and longevity in a natural environment [13]. Only males with a full sibling in the opposite treatment were included in the analyses (Total of 9 dams, 19 males in cold, 17 males in warm). Females continue to grow after sexual maturity and were therefore photographed twice after sexual maturity to estimate adult growth rates, which were shown previously to influence mate preferences [21]. Adult females (total of 42, 22 in cold, 20 in warm) were isolated into individual tanks in a room with intermediate temperature from the treatment temperatures (23°C) which avoids any confounding influence of current temperature on adult growth rates. The first size measurements of the females were made when they were initially moved to the intermediate temperature. Females from the warm treatment were significantly larger (25.7 mm, SD = 1.5) than the females from the cold treatment (23.2 mm, SD 3.71; t = -2.9, df = 40, P = 0.006) in this initial measure. The second measurement of female size was made immediately prior to testing mate preferences. In the second measurement, females from the cold treatment (mean = 38.3 mm, SD = 2.61) were significantly larger than the females from the warm treatment (mean = 35.7 mm, SD = 2.67, t = 3.212, df = 40, P = 0.003). The average number of days between the two measurements, used to assess growth rates, was not significantly different between treatments (cold mean = 340 days, SD = 72; warm mean = 361 days, SD = 44; t = -1.12, df = 40, P = 0.13). Photographs were analyzed for standard length (SL) in the measurement program ImageJ [28].

### Female mate preference tests

Previous work comparing the mate preferences of virgins and mated females in swordtails found weak or no preferences in virgin females [19,21], a trend that is not common across taxa [29]. Therefore, we tested females for their preferences for male body size after receiving mating experience. Raised to sexual maturity in the environmental chambers, virgin females were placed in an acclimation room that remained at an intermediate temperature of 23°C, between the cold (20°C) and warm (25°C) treatments. By holding all females in the same temperature for preference testing, any influence of temperature on activity levels that would confound measurements of preference were removed. After 7–12 days of acclimation, the females were moved to one of six 38-liter experience tanks. Each tank contained a large male (range of SL 34.2–37.3 mm), a male that was 2–5.5 mm smaller (range of SL 28.4–35.4 mm), and another “companion” female. The companion females were significantly smaller, to distinguish them from the females being tested. All fish used in the experience tanks (both males and companion female) were taken from courter mesocosms. In addition to only using coaxing courtship behavior regardless of male size, courter males have not been observed to be aggressive towards females in this species in the either the laboratory or the field. After seven days of experience, females were removed from the experience tank and tested immediately for their mate preference for male size.

**Fig 1:**
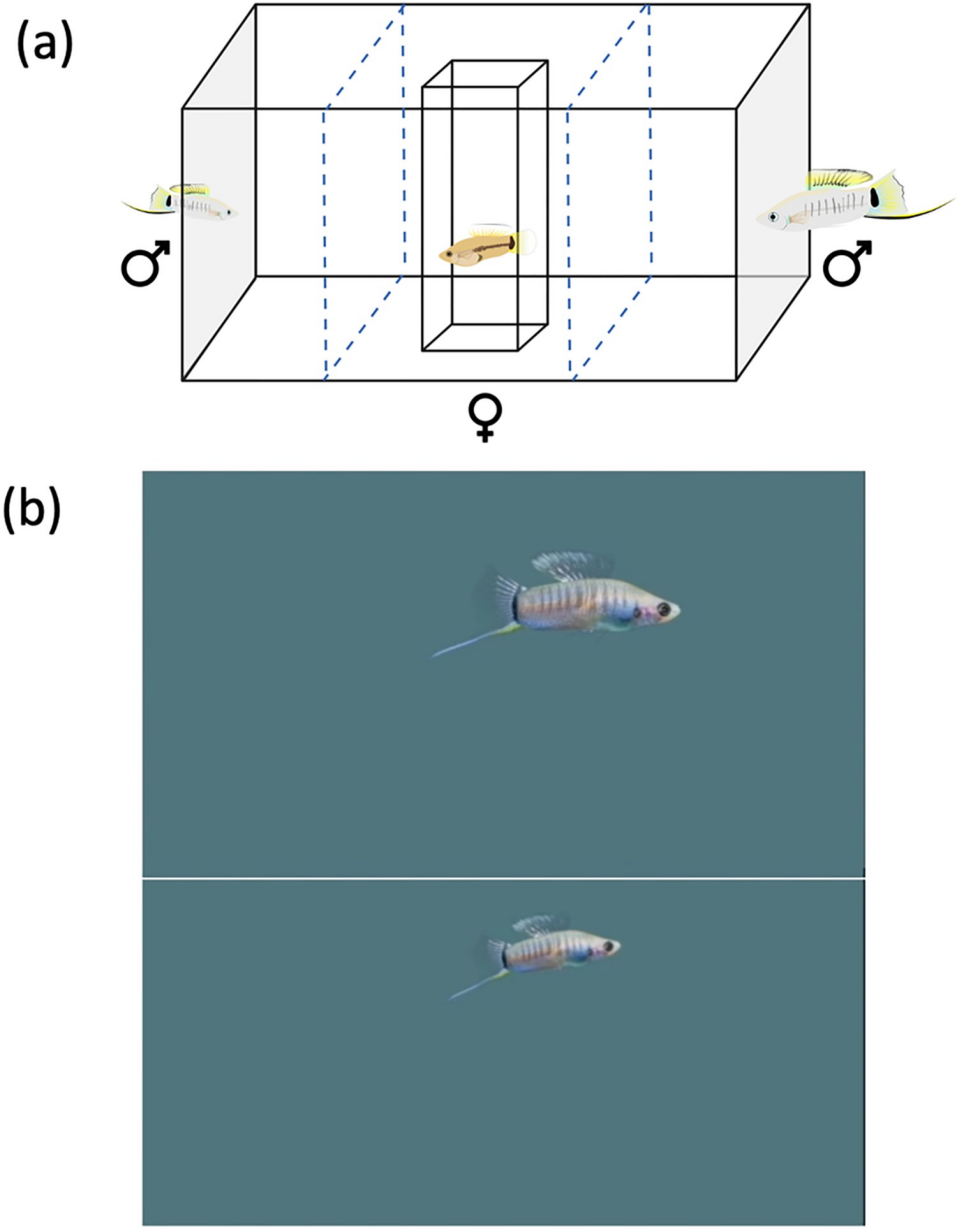
Test design. (a) Female mate preferences were tested using video animations, in dichotomous choice tests. (b) Photos of the video animations used for female mate preference testing. The upper panel shows the video image for the larger male, and the lower panel the video image for the smaller male. Both videos were modeled after the same *X*. *multilineatus* courter male. Animator: Dylan Keim.

All preference tests were conducted between 9:00–14:00. Females raised in the 25°C environmental chamber (warm) were tested first because they reached sexual maturity before females from the 20°C (cold) chamber. Preference tests were conducted in a 38-liter tank lined with gravel, with 32-inch monitors on either side of the tank (Fig 1A). Test subjects were presented video animations of males that varied only in the trait of interest (body size), which eliminates any variation in results due to male behavior or other traits that might influence mate preferences (Fig 1B). This was accomplished by manipulating the overall size of a courter male from a video animation. The swimming patterns of the fish were identical, and the relative size of both fish in each animation remained constant. The size of the male in the large video had a standard length (SL) of 39 mm and the SL of the male in the small video was 29 mm. Courter male SL varies from 22 to 42 mm in wild-caught samples [10]. The background of the animation remained a neutral blue color for the entirety of the video. The temperature of the testing tank water matched the temperature in which females were given experience. StressCoat^TM^ was added to the tank between tests to eliminate any possible chemical stress cues in the water. Tank water was fully changed twice over the duration of all the testing, in addition to topping off the tank water between tests if water levels were low due to evaporation.

After a 10-minute acclimation period in a transparent tube, the female was allowed to view the video animations for 270 seconds before being released. Time spent in the portion of the tank nearest the two animations over 300 seconds was recorded. To eliminate side biases, the trial was immediately repeated with the video animations shown on opposite sides. Two females were removed from analyses due to side biases (i.e., spent all their time on the same side of the tank across both trials).

### Statistical analyses

For all statistical models we analyzed the distribution of residuals to confirm a normal distribution. Variation in male adult size (at sexual maturity, when males stop growing) was examined using a General Linear Model (LM), with treatment (warm versus cold) nested within dam (estimate of genotype as siblings) so that we could examine the influence of treatment on male size while controlling for any possible effect of dams and testing for the homogeneity of variances between treatments with a Levene’s test in the context of the general linear model described above. The model also included early male growth rate as a covariate since this has been shown previously to affect adult male size [14; see Supporting Information for full linear model formula, SPSS subroutine and script].

We calculated absolute strength of preference as absolute difference in the total time spent with the larger male video and the total time spent with the smaller male video to compare across treatment. Given that not all the females from the two treatments spent more time with the same video, this measure provides a better indicator of female mating preferences based on male size to compare across treatments. Variation in the absolute strength of preference (SOP) was examined with a Generalized Linear Mixed Model (GLMM), with a Poisson probability distribution and a Log link function since that resulted in the normal distribution of the residuals. The model included dam as a random factor, female growth rate as a covariate and its interaction with treatment (see Supporting Information for full linear model formula, SPSS subroutine and script). We included growth rates in our analyses of the variation in strength of preference as we detected significant differences in the post maturation growth rates for females from the two rearing treatments measured while held in the intermediate temperature (Cold = 0.044 mm/day, Warm = 0.028, t = 6.09, df = 40, P = 0.001). Additionally, female growth rates have been demonstrated to influence variation in strength of preference for males from the two alternative reproductive tactics that differ in male size in this species [21]. Finally, we did an alternative analysis of SOP using female body size (SL prior to testing for mate preference) rather than growth rates as covariate and we report the results from this alternative analysis in the Supporting Information. There was no difference between treatments in the total time spent associating with the two videos (cold mean = 427.3, warm mean = 455.6, t = -1.31, df = 41, P = 0.19) and therefore this factor was not included in the model. Pairwise contrasts of SOP for the two treatments were examined with a sequential Bonferroni adjustment, and we report the estimated marginal means for the absolute SOP. We also examined preference within treatments comparing total time with larger male video and smaller male video with paired t-tests [30].

The signal reliability hypothesis predicts that strength of preference would be greater for females reared in the condition where variation in male size would be greatest across genotypes (i.e., dams). We analyzed a total of 22 females from the cold and 20 females from the warm treatment. All analyses were conducted in the program IBM® SPSS® Statistics Version 25.

## Results

### Male size across treatments

**Fig 2:**
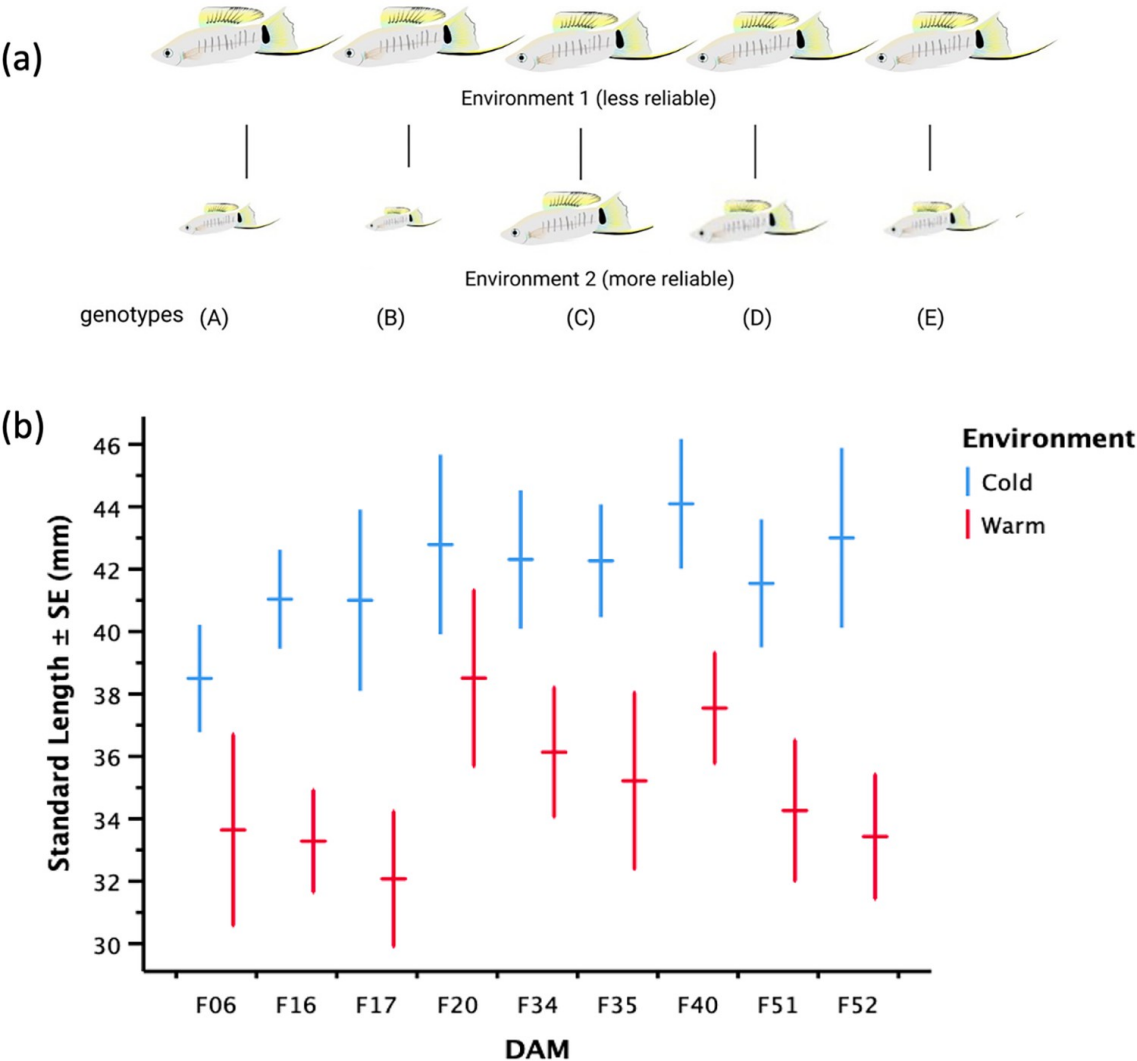
Signal reliability hypothesis. (a) The signal reliability hypothesis suggests that for a conditionally influenced male trait like body size, male size would be a better indicator of any genetic influence (i.e., more reliable) in condition 2 as compared to condition 1. (b) Estimated means of male size at sexual maturity (± Standard Error), when *X*. *multilineatus* males stop growing [11], was influenced by environment (temperature treatment), with size being larger in the cold (blue) but significantly more variable and therefore more reliable in the warm environment (red), controlling for dam.

We did not find a significant effect of growth rate on male size (LM: *F*_1, 17_ = 2.73, *P* = 0.12); however, we did find that male size across the courter ART males was significantly influenced by rearing temperature (LM: *F*_1, 17_ = 3.18, *P* = 0.01), and variances in male sizes were not equal across rearing temperatures (Levene’s Test of Equality of Variances: Levene Statistic _17, 18_ = 3.19, *P* = 0.01). The signal reliability hypothesis suggests that traits like male size may provide more information about a male’s genotype in some environments as compared to others, with variation across genotypes greater in the environment as an indicator of more information (Fig 2A). There was significantly more variation across dams in the warm (Variance = 12.22) than the cold environment (Variance 11.94; Fig 2B). Therefore, male size would be a more reliable indicator of male genotype in a warm as compared to a cold environment.

### Female mate preferences

**Fig 3:**
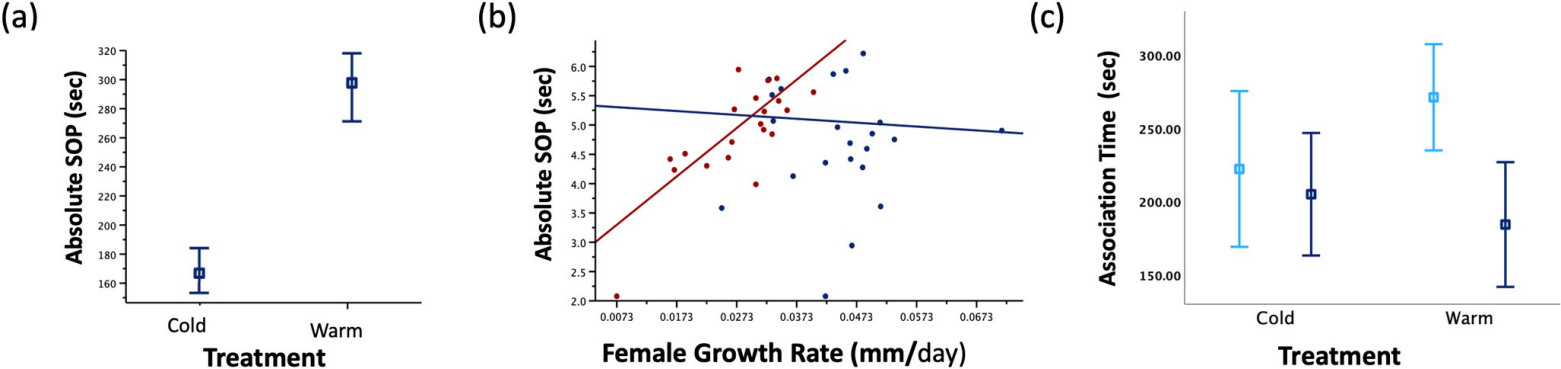
Preference test results. (a) Significantly greater absolute SOP for females reared in the warm environment (estimated marginal means ± standard errors) as compared to their siblings reared in the cold environment. (b) Female’s growth rate after sexual maturity influenced absolute strength of preference (plotted as natural logarithm, see methods); females from the warm environment (red circles) had a stronger absolute strength of preference (SOP) if they grew faster, a pattern not detected in females from the cold environment (blue circles); (c) Females from the warm environment had a significant preference for the smaller male video (light blue) as compared to the larger male video (dark blue); no preference was detected in their siblings reared in the cold environment.

Absolute SOP for male size was influenced by rearing temperature (GLMM: *F*_1, 38_ = 304.65, *P* < 0.001), growth (GLMM: *F*_1, 38_ = 283.05, *P* < 0.001) and an interaction between treatment and growth rate (GLMM: *F*_1, 38_ = 411.34, *P* < 0.001). Females reared in the warm environment had an overall greater absolute strength of preference than females reared in the cold environment (Fig 3A). Females from the warm environment had a stronger SOP if they grew faster (Fig 3B), but that was not the case for females from the cold environment. A Wald test for the covariance of the random effect parameter showed that the effect of the dam on SOP was significant (Z = 2.37; *P* = 0.01). Finally, females from the warm treatment had a significant preference for smaller males (t = -2.16, P = 0.04) that was not detected for females from the cold treatment (t = 0.38, P = 0.70, Fig 3C).

### Discussion

We present support for the signal reliability hypothesis as females had a stronger mate preference for male size when reared in an environment where male size would be a better indicator of genotype. Males in the cold treatment were larger than their siblings in the warm, and therefore the greater variation in size in the warm is not simply due to larger sizes. It has been well established that both sexually selected traits and preferences for these traits can be context dependent [31,32]. However, what has not been studied extensively is the extent to which the condition dependence of male traits leading to more or less reliable signals across contexts could select for stronger preferences in some contexts. Given that the influence of genetic variation on the phenotypic expression of traits will be greater in some context than others [9], testing for differences in preferences for these traits across the same contexts is a logical next step for understanding adaptive variation in mate preferences.

Previous studies of mate preferences in this species detected preferences for males from the courter alternative reproductive tactic (ART) as compared to the on average smaller sneaker ART males [10,16,33]. Given that the males from the two alternative reproductive tactics are dimorphic for morphological and behavioral traits in addition to body size, in the current study we controlled for ART by giving females a choice between a large and small courter male video, controlling for differences other than overall size. The females reared in the warm treatment had an overall preference for the smaller courter male as compared to the larger courter male, suggesting potential selection in this species for the intermediate sized males. Future studies will want to disentangle preference for body size from preference for other traits that differ between the ARTs before examining selection on male size, as well as potential information male size provides females in this species.

Hypotheses for the evolution of mate preferences for size are numerous, as is the evidence for variation in mate preferences for size [34]. Studies have found that preferences for size can vary based on environmental conditions such as predation (e.g., [35]) and the condition of the chooser (e.g., [36,37]). Preferences can also be assortative based on both individuals’ condition (e.g., [38]). These results, and many others, have called into question the idea that preference for size evolved as a sensory bias or by Fisher’s Runaway, with no subsequent selection due to the benefits of preference [34]. Additionally, theoretical work supports the prospect of the mechanisms of indirect genetic benefits and sensory bias co-occurring [39], as well as benefits of indirect and direct selection being equally as strong [40]. The current study adds to the hypotheses for the evolution of mate preferences for male size based on indirect benefits by supporting a previously suggested hypothesis of potential disassortative mating based on growth rates. However, the current study also suggests that if male size provides females with information about genetic influences on male growth rates, this information may only be available in some contexts.

The hypothesis for disassortative mating based on growth rates was first presented in a previous study of variation in mate preferences in *X*. *multilineatus* [21]. A female’s genotype (courter versus sneaker sire) influenced their strength of preference for the larger courter males as compared to smaller sneaker males, with females from the courter lineage that grow faster [41] having a weaker preference for the courter males that grow faster [21]. A relationship between adult growth rate and strength of preference within the sneaker lineage females was also detected in this previous study [21]. In the current study we did not find an influence of growth rates on male adult size, however, a relationship between male growth rate and age/size at sexual maturity has been detected previously in a field study of this species [11]. If male size provides information about genetic influences on growth rates, females could be under selection to mate with males with dissimilar growth rates, thus optimizing the growth-mortality tradeoff [42] for their offspring. The growth-mortality tradeoff (higher growth rate increases probability of reaching sexual maturity, but also results in shorter-lived adults) has been documented for this species in both laboratory [14] and field studies [13]. The strength of mate preference for smaller males by female from the warm treatment detected in the current study was correlated with the growth rates of these females, suggesting temperature may be a mechanism that links growth rate with preference. In addition, dam was a significant factor influencing strength of preference, suggesting a genetic influence and further supporting the potential for disassortative mating based on growth rates. An alternative hypothesis for the variation in preferences within the warm reared females is size assortative mating, a pattern that has been detected in other fish species [43]. However, we did not find a significant influence of female size on strength of preference (see Supporting Information), and currently have no explanation for the benefits of disassortative mating based on body size in this species. In either case, it is interesting to note that temperature has been shown to influence the signal-preference relationship in other systems [43]. Future studies of the gene by environmental temperature influences on growth rates, as well as the fitness benefits of mate preferences in relation to growth rates, could test the hypothesis that mate preferences for size are under selection due to male size as an indicator of growth rates in some contexts.

An outstanding question that this study cannot address is why male size was a more reliable indicator of genotype in the warm as compared to cold environment. There has been a lot of interest in the genetic influences for size/age at sexual maturity since the polymorphisms in size were first described in a *Xiphophorus* fish [44]. Lampert and colleagues demonstrated that sequence polymorphisms of *mc4r* comprise both functional and non-signal-transducing versions, with higher expression of the mutant non-signal-transducing versions due to more copies of the B allele in the larger courter males [22]. While studies of the *mc4r* system in relation to body size and puberty onset continue [23,45–47], interactions between the genetic and environmental influences have not been directly examined. Further study of the mechanisms involved in regulating growth and age at sexual maturity in this system, leading to variation in male size due to both genetics and environmental influences, will provide valuable insights into the evolution of the size variation in these fishes.

## Conclusion

Wagner argued that environmental effects on signal reliability may be widespread, leading to mate choice based on these signals requiring the evolution of complex assessment strategies [1]. We detected stronger female mate preference for male size by females reared in the same environment where male size would most reliably indicate genotype, providing initial support for an assessment strategy based on signal reliability.

## Supporting information

S1 File (DOCX)
