# Supplementary material for: Strength of female mate preferences in temperature manipulation study supports the signal reliability hypothesis
Source: PLoS One. 2024 Jun 6;19(6):e0303691. doi: 10.1371/journal.pone.0303691 (PMC11156382; doi:10.1371/journal.pone.0303691)
Supplement: S1 File — (DOCX) [file pone.0303691.s001.docx]

**Supporting Information**

**I. Statistical Analyses**

**S1 Text.**

*(a) Male size across treatments*

Full linear model: male size~Treatment(Dam) + Growth.

SPSS subroutine: UNIANOVA.

SPSS script:

UNIANOVA SL BY Treatment DAM WITH GR100
  /RANDOM=DAM
  /METHOD=SSTYPE(3)
  /INTERCEPT=INCLUDE
  /SAVE=RESID ZRESID SRESID DRESID
  /PLOT=PROFILE(Treatment) TYPE=LINE ERRORBAR=SE(1) MEANREFERENCE=NO YAXIS=AUTO
  /EMMEANS=TABLES(Treatment) WITH(GR100=MEAN)
  /PRINT DESCRIPTIVE HOMOGENEITY
  /CRITERIA=ALPHA(.05)
  /DESIGN=Treatment(DAM) GR100.

*(b) Female mate preferences*

Full linear model: model: SOP~Treatment + (|Dam) + Growth + Treatment*Growth.

SPSS subroutine: GENLINMIXED.

SPSS script:

*Generalized Linear Mixed Models.
GENLINMIXED
  /FIELDS TARGET=abssize TRIALS=NONE OFFSET=NONE
  /TARGET_OPTIONS DISTRIBUTION=POISSON LINK=LOG
  /FIXED  EFFECTS=Treatment growth Treatment*growth USE_INTERCEPT=TRUE
  /RANDOM EFFECTS=Mom USE_INTERCEPT=FALSE COVARIANCE_TYPE=VARIANCE_COMPONENTS SOLUTION=FALSE
  /BUILD_OPTIONS TARGET_CATEGORY_ORDER=ASCENDING INPUTS_CATEGORY_ORDER=ASCENDING MAX_ITERATIONS=100 CONFIDENCE_LEVEL=95 DF_METHOD=RESIDUAL COVB=MODEL PCONVERGE=0.000001(ABSOLUTE) SCORING=0 SINGULAR=0.000000000001
  /EMMEANS TABLES=Treatment COMPARE=Treatment CONTRAST=PAIRWISE
  /EMMEANS_OPTIONS SCALE=ORIGINAL PADJUST=SEQBONFERRONI.

**II. Individual Data Presentation**

**
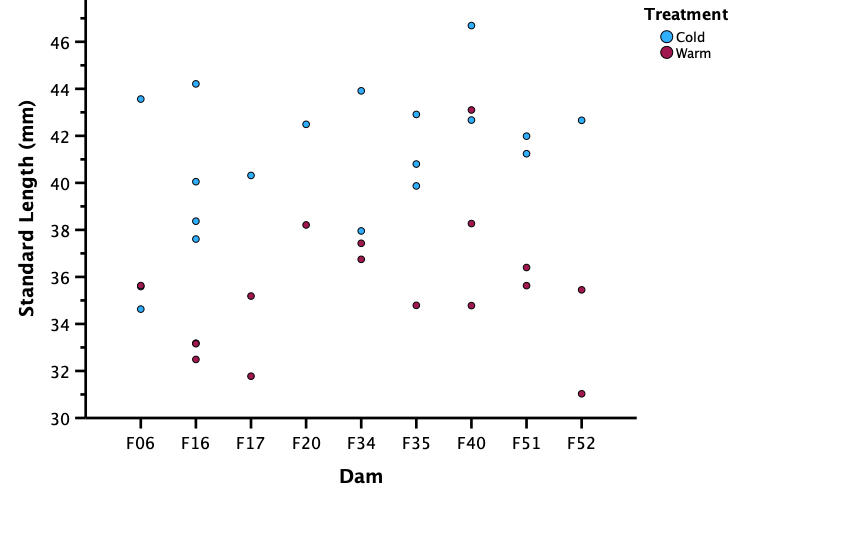
**

**S1 Fig.** Male size at sexual maturity for siblings placed in two different temperature treatments.

**
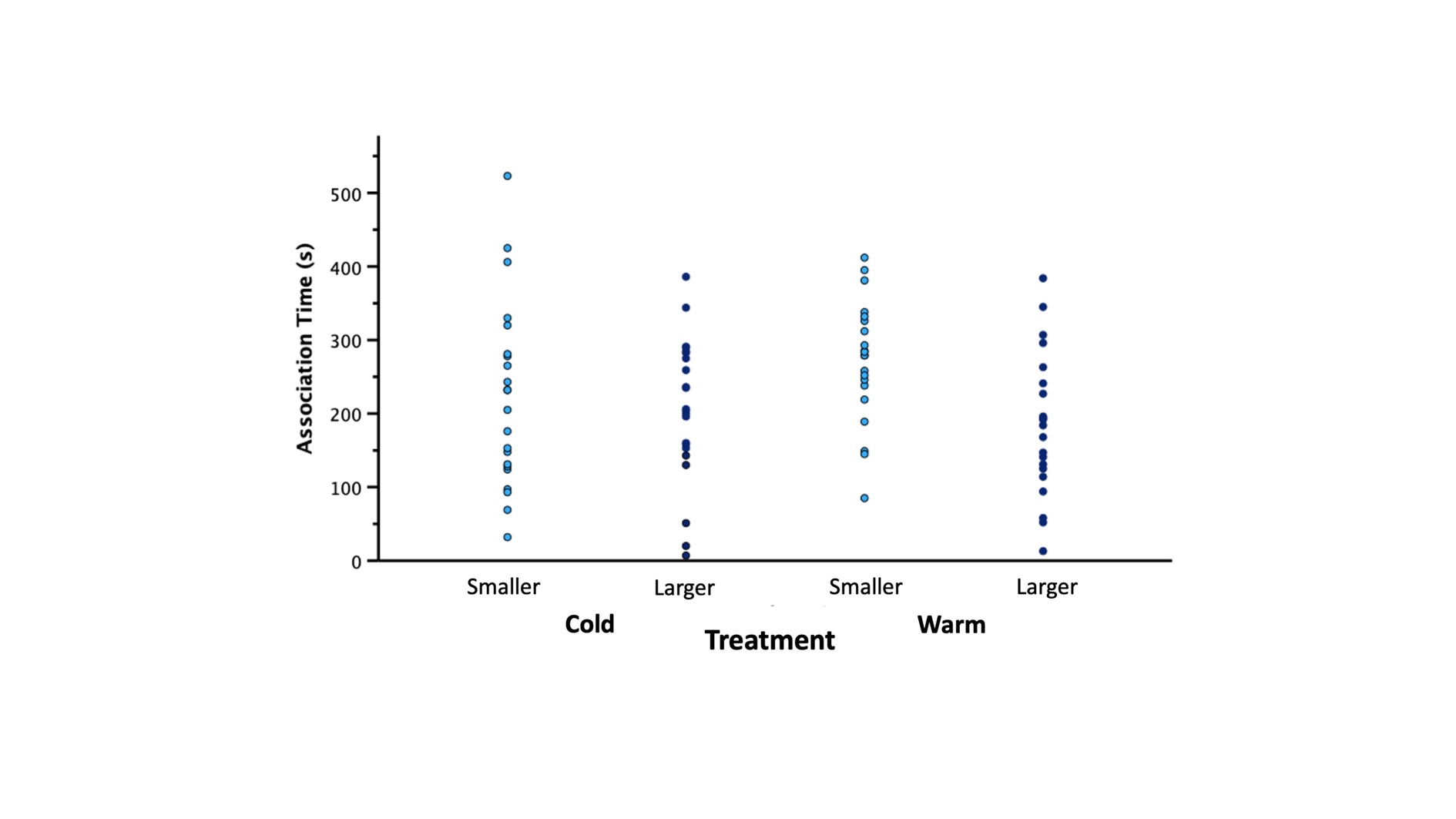
**

**S2 Fig.** Data points for individual females of time spent with video of the smaller male (light blue) as compared to time spent with the video of the larger male (dark blue). Females from the warm spent significantly more time with the video of the smaller male, but no significant difference was found for the females reared in the cold treatment (see main text).

**III. Alternative Analyses**

**S2 Text**. *Female mate preferences (with female body size as covariate)*

The results from this alternative analysis were very similar to the original one that used growth rate as covariate (see main text). Absolute SOP for male size was influenced by rearing temperature (GLMM: *F*_1, 38_ = 32.28, *P* < 0.001), and an interaction between treatment and body size (GLMM: *F*_1, 38_ = 33.56, *P* < 0.001). However, in this case, there was no significant overall effect of body size (GLMM: *F*_1, 38_ = 1.7, *P* = 0.2). Females reared in the warm environment had an overall greater absolute strength of preference than females reared in the cold environment (figure 3a). Females from the warm environment had a stronger SOP if they were larger, but that was not the case for females from the cold environment (figure 3b). A Wald test for the covariance of the random effect parameter showed that the effect of the dam on SOP was significant (Z = 2.38; *P* = 0.017).

**
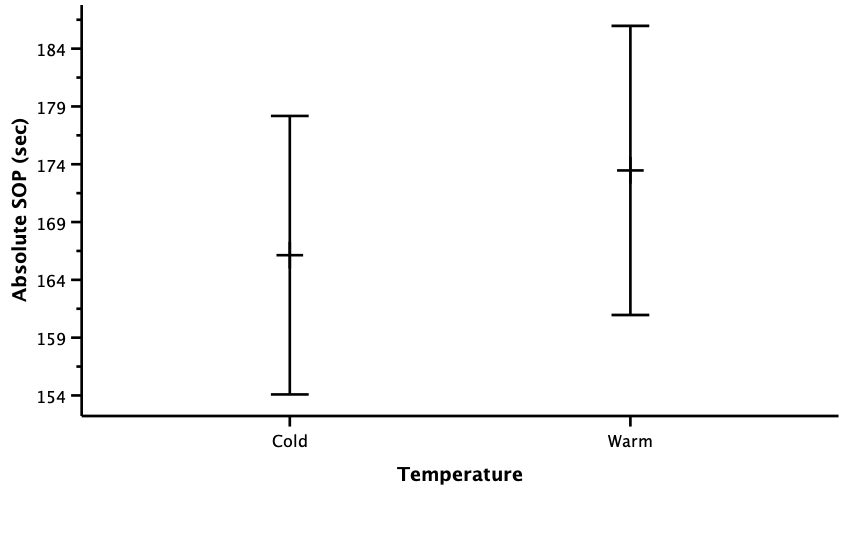
**

(a)

**
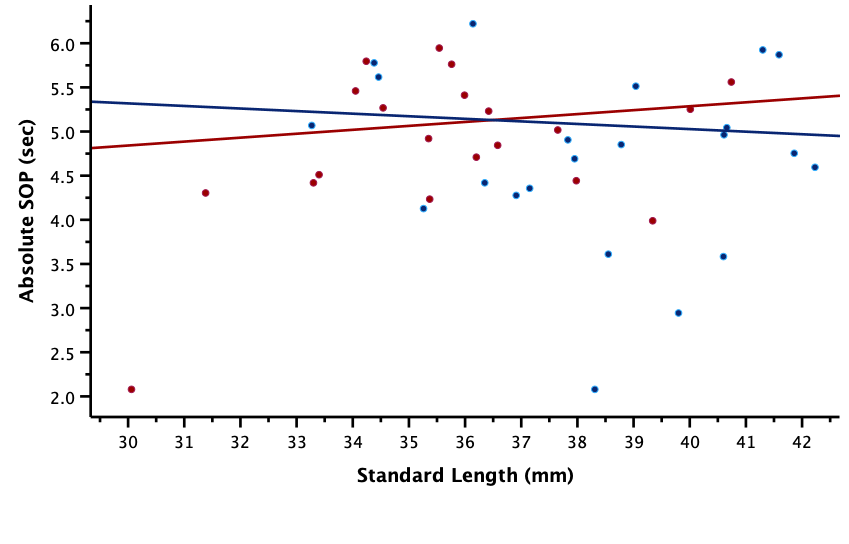
**

(b)

**S3 Fig.** Preference test alternative results. (a) Significantly greater absolute SOP for females reared in the warm environment (estimated marginal means ± standard errors) as compared to their siblings reared in the cold environment. (b) The effect of female’s body size after sexual maturity on absolute strength of preference (plotted as natural logarithm, see methods) was different between treatments; females from the warm environment (red circles) had a stronger absolute strength of preference (SOP) if they were larger, the opposite effect was detected in females from the cold environment (blue circles).

**S3 Text.**

*Female growth rates and body size*

In addition, we explored the relation between body size and growth rate and found that there was a significant relation between these two variables (Linear regression: *F*_1, 40_ = 25.23, *P* < 0.001).

*Model comparisons*

Finally, we compared the original model (see main text) and the one presented here (see above) using Akaike’s information criterion corrected for small sample size (AICc) and the coefficient of determination (Pseudo-R^2^). The model that used growth rate as covariate fit better than the one with body size (growth rate: AICc =2144.03, Pseudo-R^2^ = 0.67; body size: AICc = 2813.25, Pseudo-R^2^ = 0.12).

Results from the models’ comparison were not surprising since we had detected the effect of growth rate on female mate preferences in previous studies (see main text). Therefore, to avoid any collinearity effects from the correlation between these two covariates, we decided to use the one that gave a model with a better fit to the data. Nevertheless, the lack of a significant overall effect of body size on mate preference suggests that the pattern we are detecting is not simply the result of assortative mating for body size.
